# Supplementary material for: Curcumin Treatment Identifies Therapeutic Targets within Biomarkers of Liver Colonization by Highly Invasive Mesothelioma Cells—Potential Links with Sarcomas
Source: Cancers (Basel). 2020 Nov 16;12(11):3384. doi: 10.3390/cancers12113384 (PMC7696465; doi:10.3390/cancers12113384)
Supplement: Supplementary file 1 [file cancers-12-03384-s001.zip › cancers-965878-supplementary.pdf]

Supplementary Materials

Curcumin Treatment Identifies Therapeutic Targets within Biomarkers of Liver Colonization by Highly Invasive Mesothelioma Cells — Potential Links with Sarcomas

Daniel L. Pouliquen, Alice Boissard, Cécile Henry, Stéphanie Blandin, Pascal Richomme, Olivier Coqueret and Catherine Guette

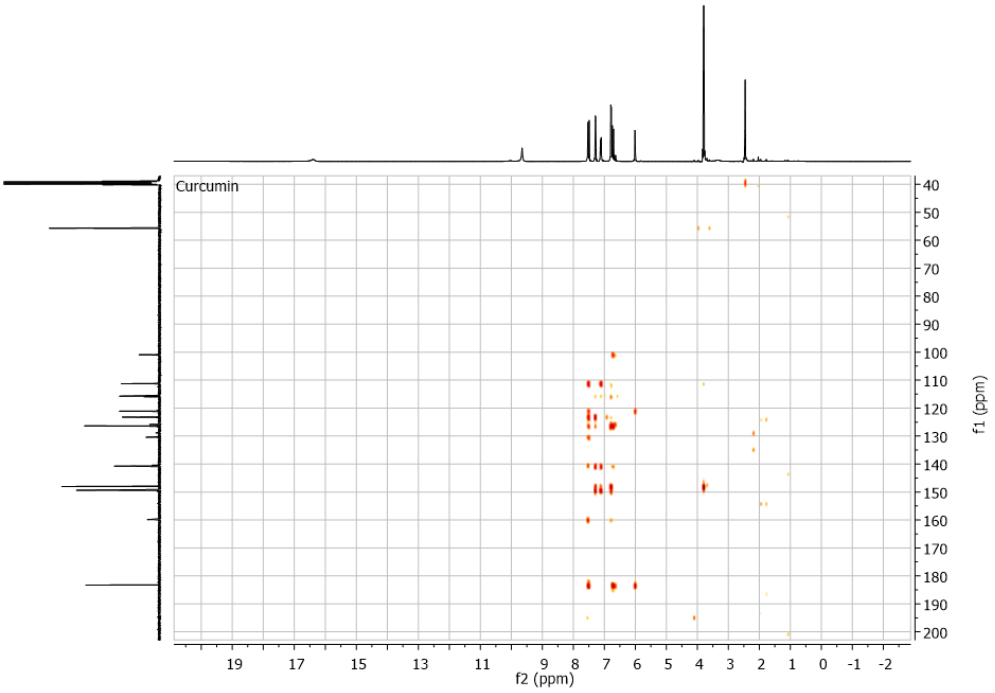

Figure S1. NMR spectra of the batch of curcumin used to treat the G3 group of rats. The 2D HMBC (^{1}H observed heteronuclear long-range J_{HC} correlations) was run using the standard JEOL pulse sequence (hmbc.jxp, 32 scans) on a JEOL 400-MHz YH spectrometer at 298 K.

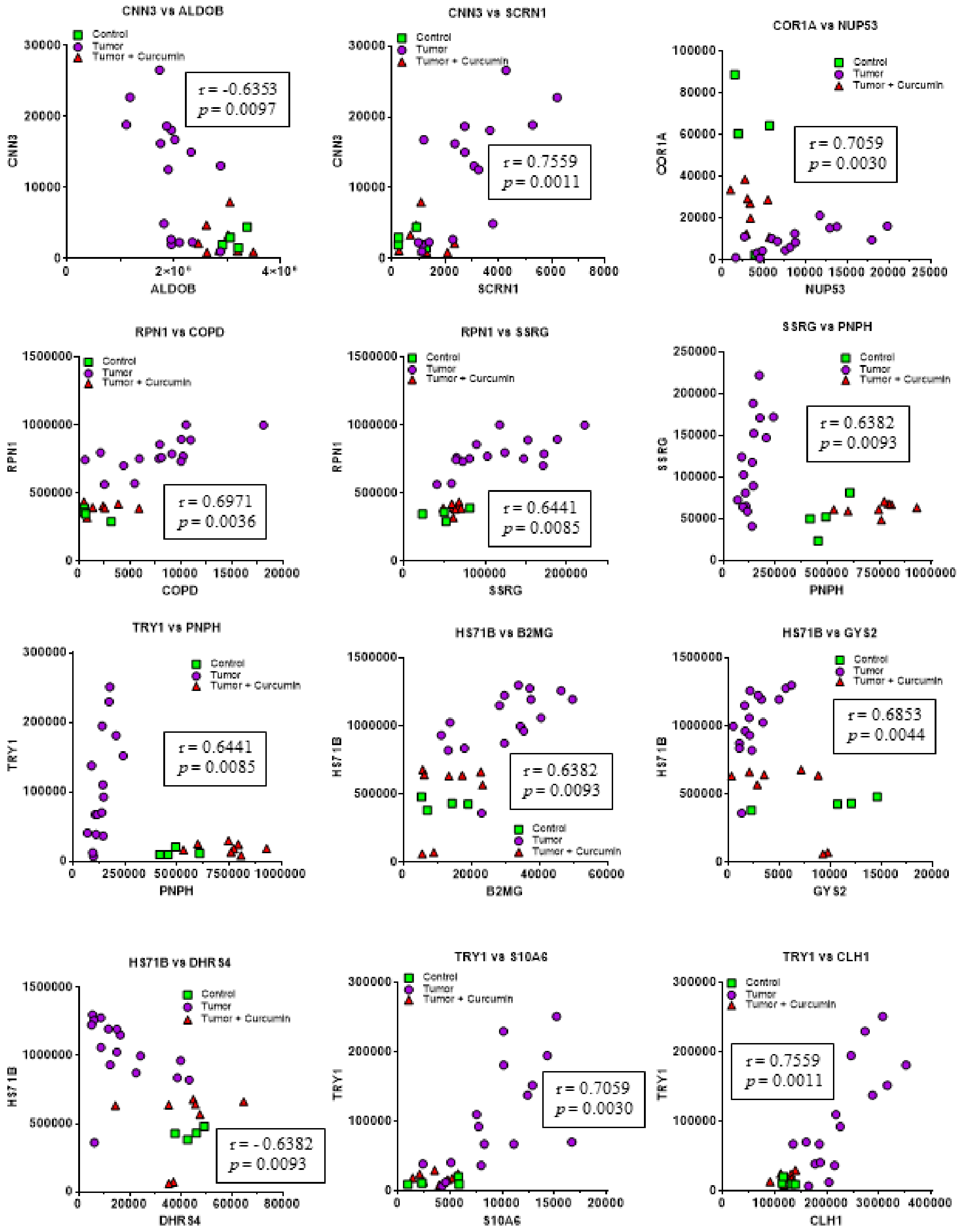
CNN3 vs ALDOB
r = -0.6353
p = 0.0097
CNN3 vs SCRN1
r = 0.7559
p = 0.0011
COR1A vs NUP53
r = 0.7059
p = 0.0030
RPN1 vs COPD
r = 0.6971
p = 0.0036
RPN1 vs SSRG
r = 0.6441
p = 0.0085
SSRG vs PNPH
r = 0.6382
p = 0.0093
TRY1 vs PNPH
r = 0.6441
p = 0.0085
HS71B vs B2MG
r = 0.6382
p = 0.0093
HS71B vs GYS2
r = 0.6853
p = 0.0044
HS71B vs DHRS4
r = -0.6382
p = 0.0093
TRY1 vs S10A6
r = 0.7059
p = 0.0030
TRY1 vs CLH1
r = 0.7559
p = 0.0011
Control
Tumor
Tumor + Curcumin

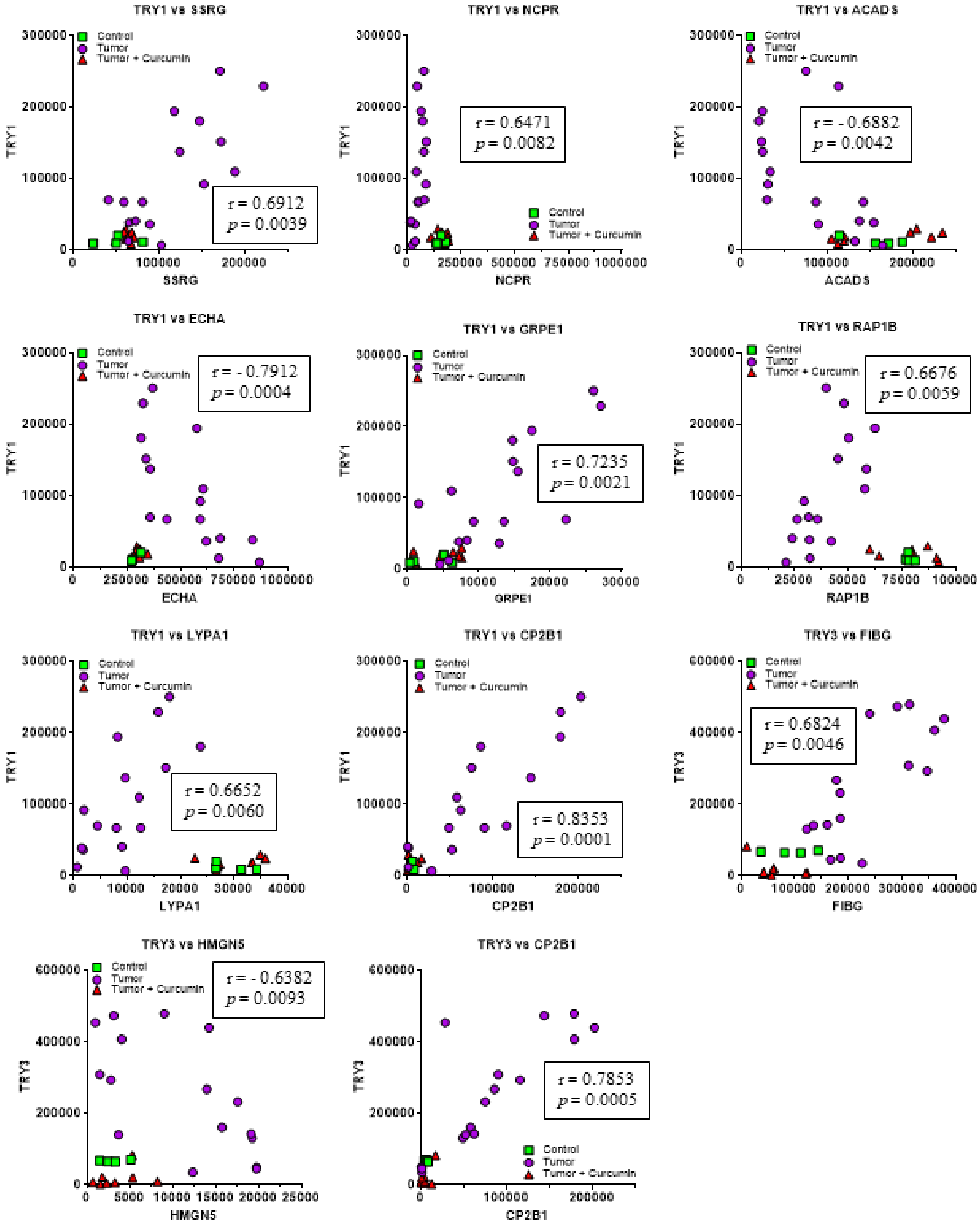

Figure S2. Correlation plots, $p < 0.01$. Correlations between biomarkers identified in sections 2.2.–2.4. and 2.5. p- and r-values obtained for statistical analysis using a Spearman's test (provided only for the group of untreated rats, purple open circles) are indicated in a rectangle in each figure.

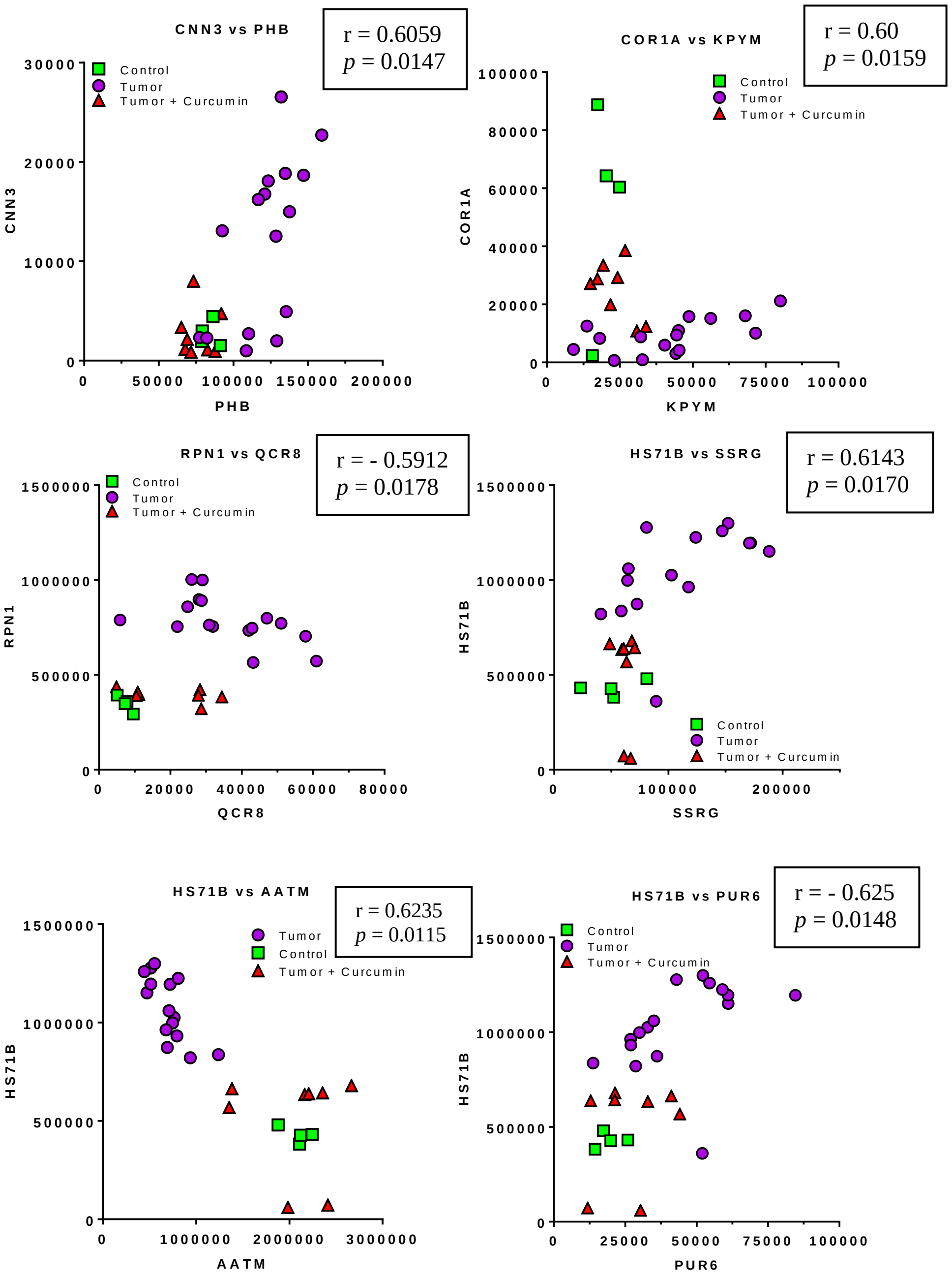
CNN3 vs PHB
r = 0.6059
p = 0.0147
Control
Tumor
Tumor + Curcumin
CNN3
PHB
COR1A vs KPYM
r = 0.60
p = 0.0159
Control
Tumor
Tumor + Curcumin
COR1A
KPYM
RPN1 vs QCR8
r = - 0.5912
p = 0.0178
Control
Tumor
Tumor + Curcumin
RPN1
QCR8
HS71B vs SSRG
r = 0.6143
p = 0.0170
Control
Tumor
Tumor + Curcumin
HS71B
SSRG
HS71B vs AATM
r = 0.6235
p = 0.0115
Tumor
Control
Tumor + Curcumin
HS71B
AATM
HS71B vs PUR6
r = - 0.625
p = 0.0148
Control
Tumor
Tumor + Curcumin
HS71B
PUR6

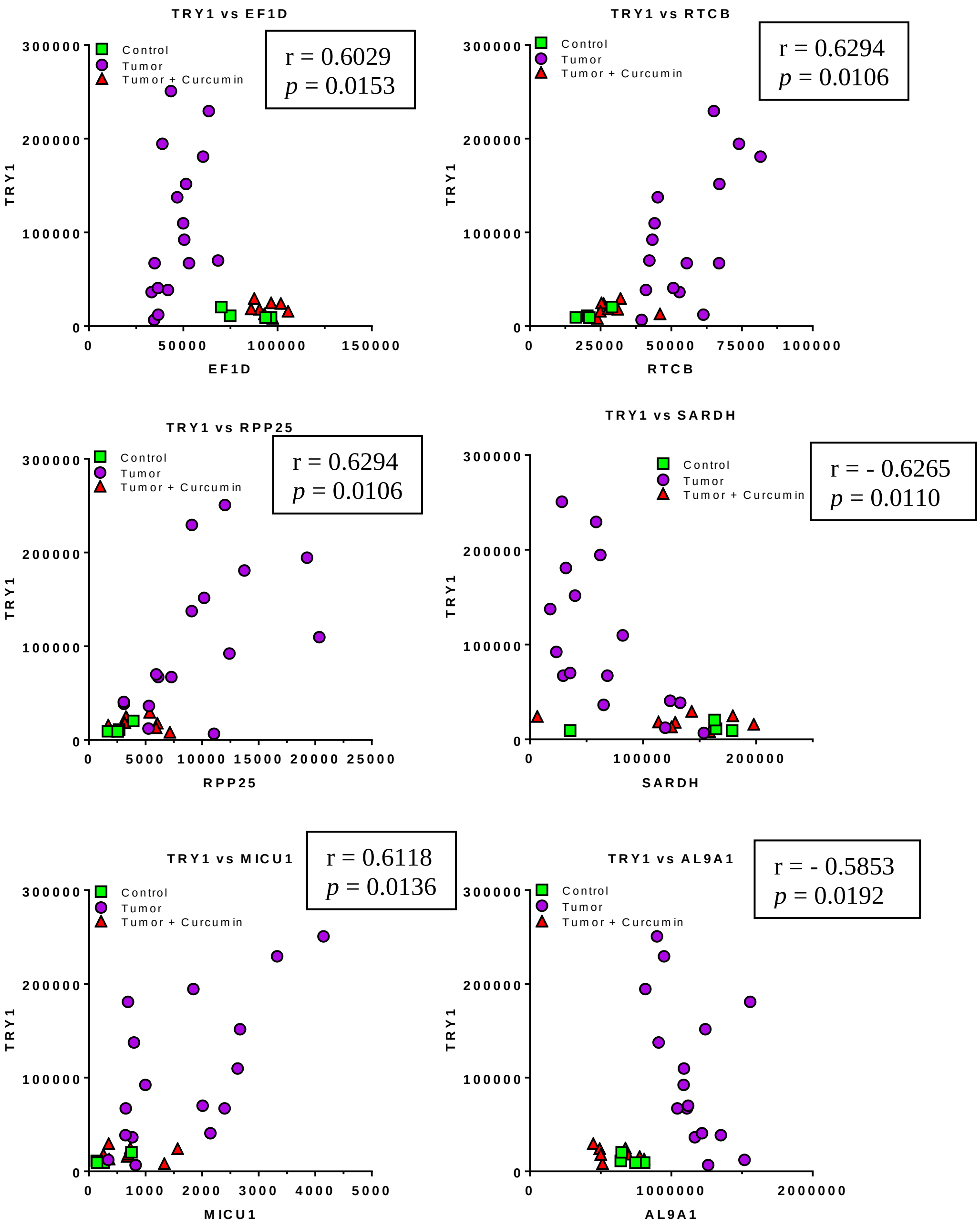

TRY1 vs EF1D
Control
Tumor
Tumor + Curcumin
r = 0.6029
p = 0.0153
300000
200000
100000
0
TRY1
0
50000
100000
150000
EF1D
TRY1 vs RTCB
Control
Tumor
Tumor + Curcumin
r = 0.6294
p = 0.0106
TRY1
0
25000
50000
75000
100000
RTCB
TRY1 vs RPP25
Control
Tumor
Tumor + Curcumin
r = 0.6294
p = 0.0106
TRY1
0
5000
10000
15000
20000
25000
RPP25
TRY1 vs SARDH
Control
Tumor
Tumor + Curcumin
r = - 0.6265
p = 0.0110
TRY1
0
100000
200000
SARDH
TRY1 vs MICU1
r = 0.6118
p = 0.0136
Control
Tumor
Tumor + Curcumin
TRY1
0
1000
2000
3000
4000
5000
MICU1
TRY1 vs AL9A1
r = - 0.5853
p = 0.0192
Control
Tumor
Tumor + Curcumin
TRY1
0
1000000
2000000
AL9A1

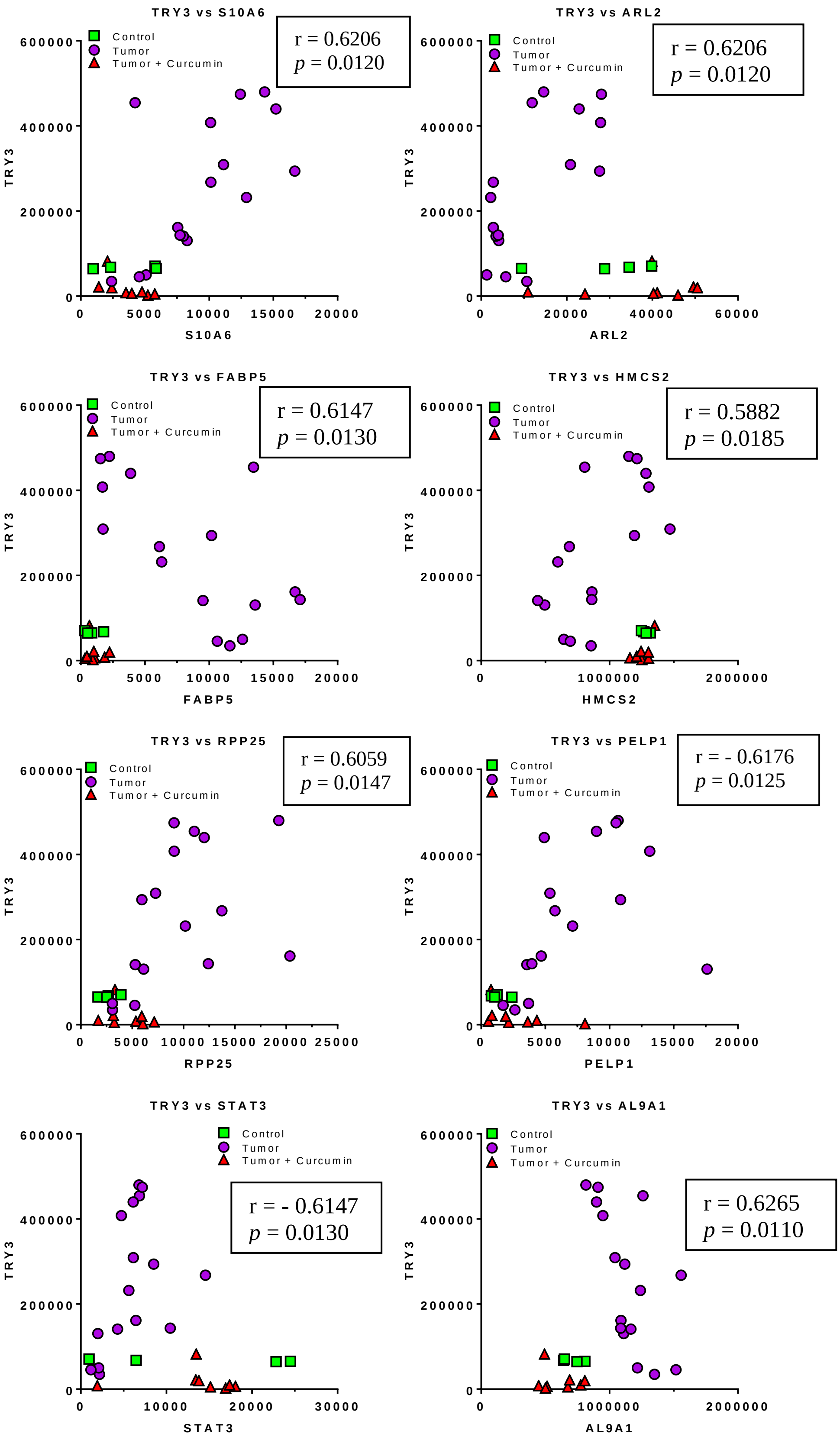

Figure S3. Correlation plots, $0.01 < p < 0.02$. Correlations between biomarkers identified in sections 2.2.–2.4. and 2.5. *p*- and *r*-values obtained for statistical analysis using a Spearman's test (provided only for the group of untreated rats, purple open circles) are indicated in a rectangle in each figure.

(1) Correlations with CNN3

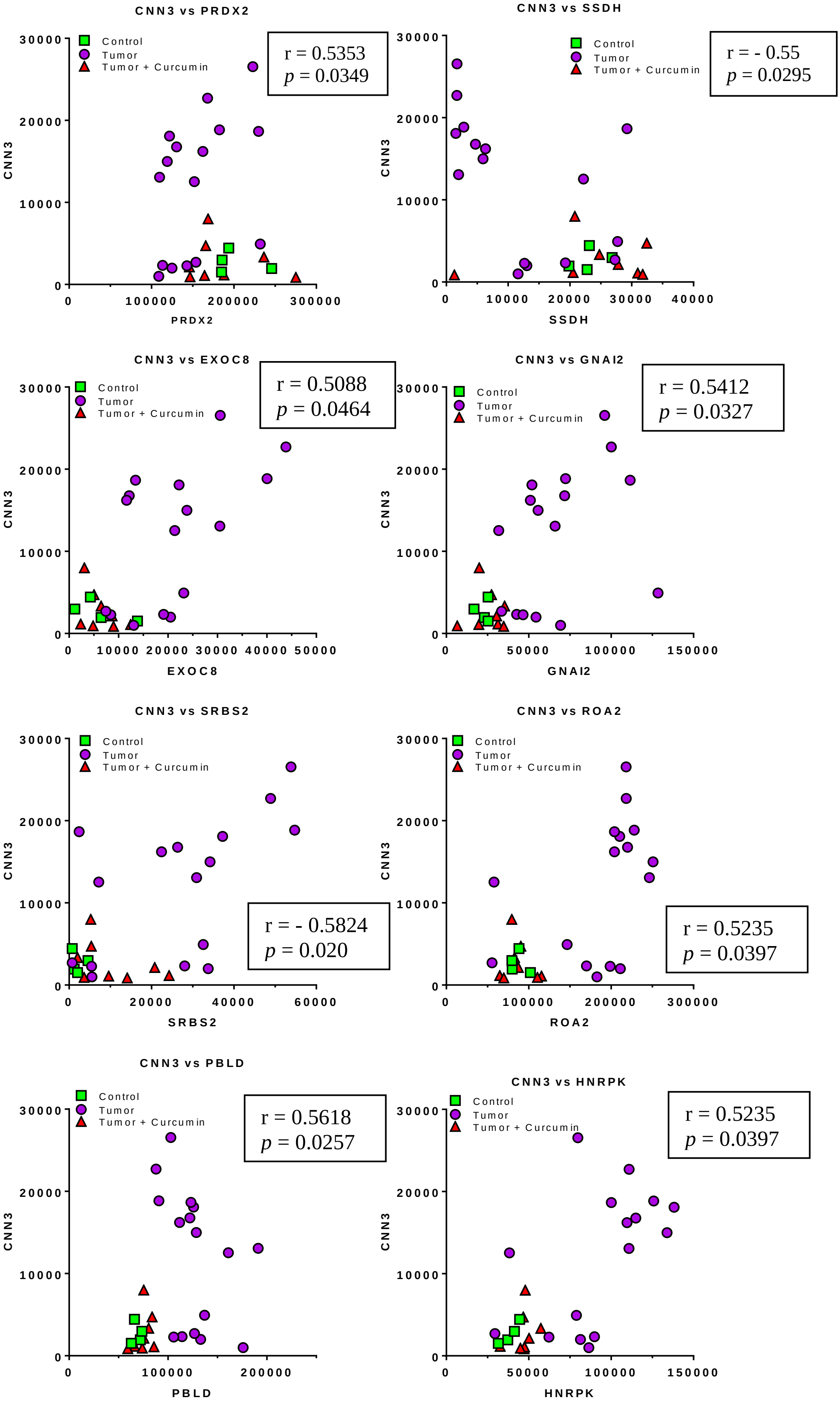

(2) Correlations with COR1A

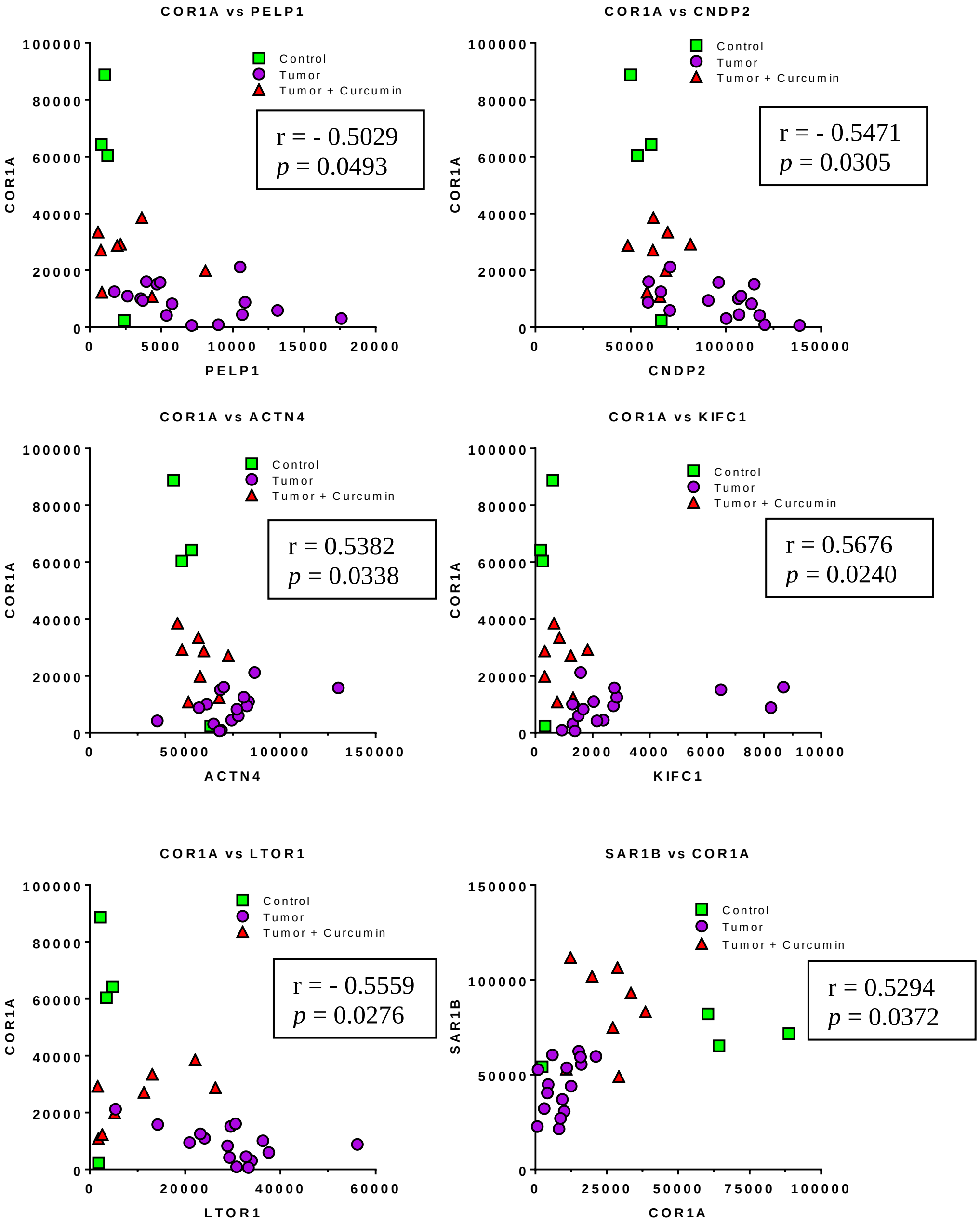

(3) Correlations with RPN1

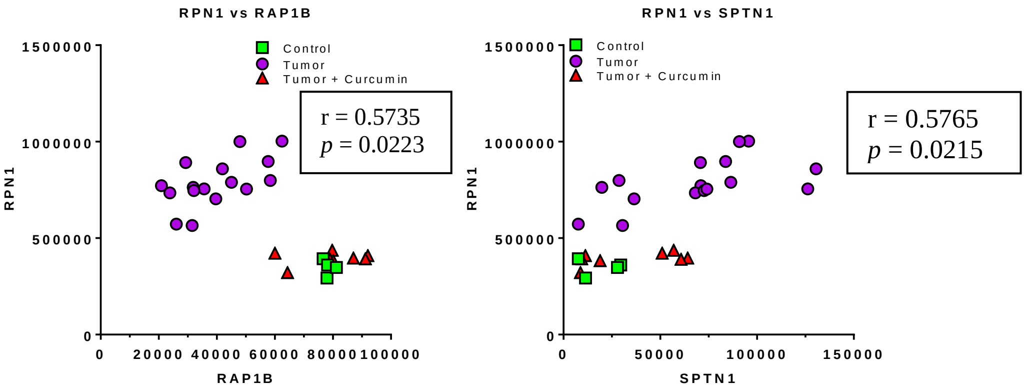

(4) Correlations with PNPH

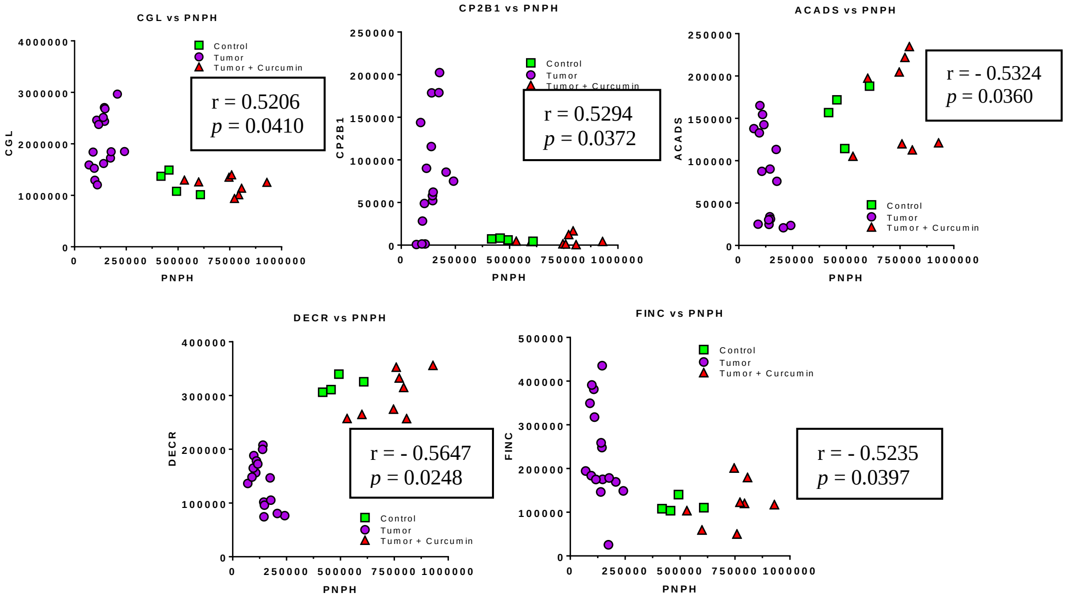

(5) Correlations with SMAD3

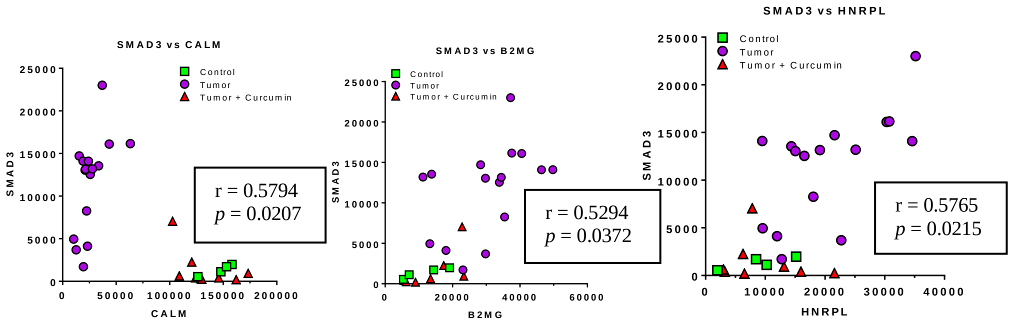

(6) Correlations with HS71B

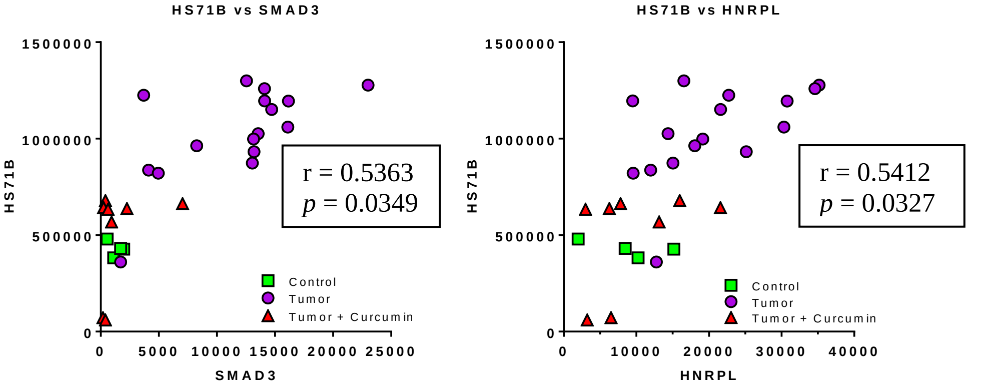

(7) Correlations with TRY1

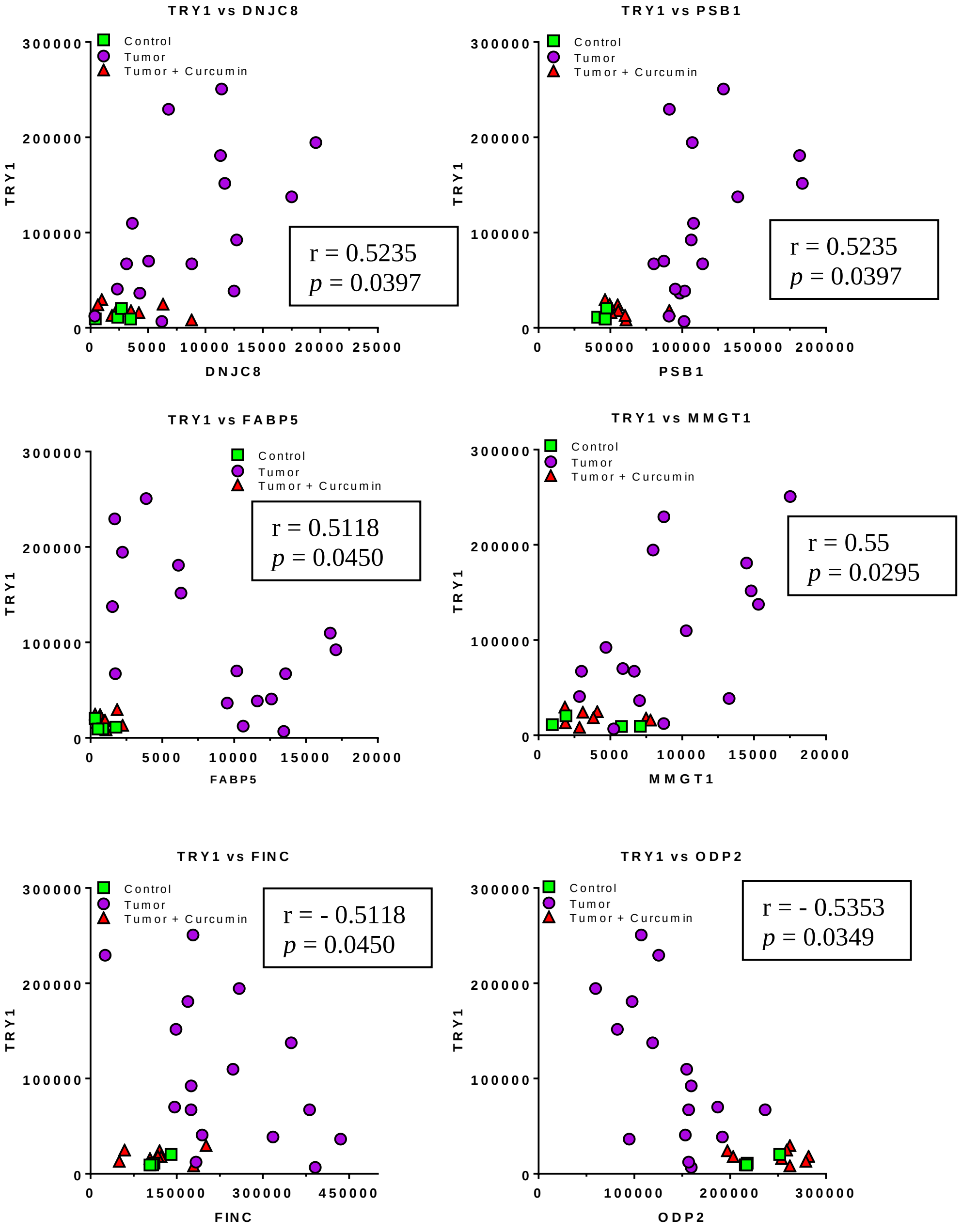

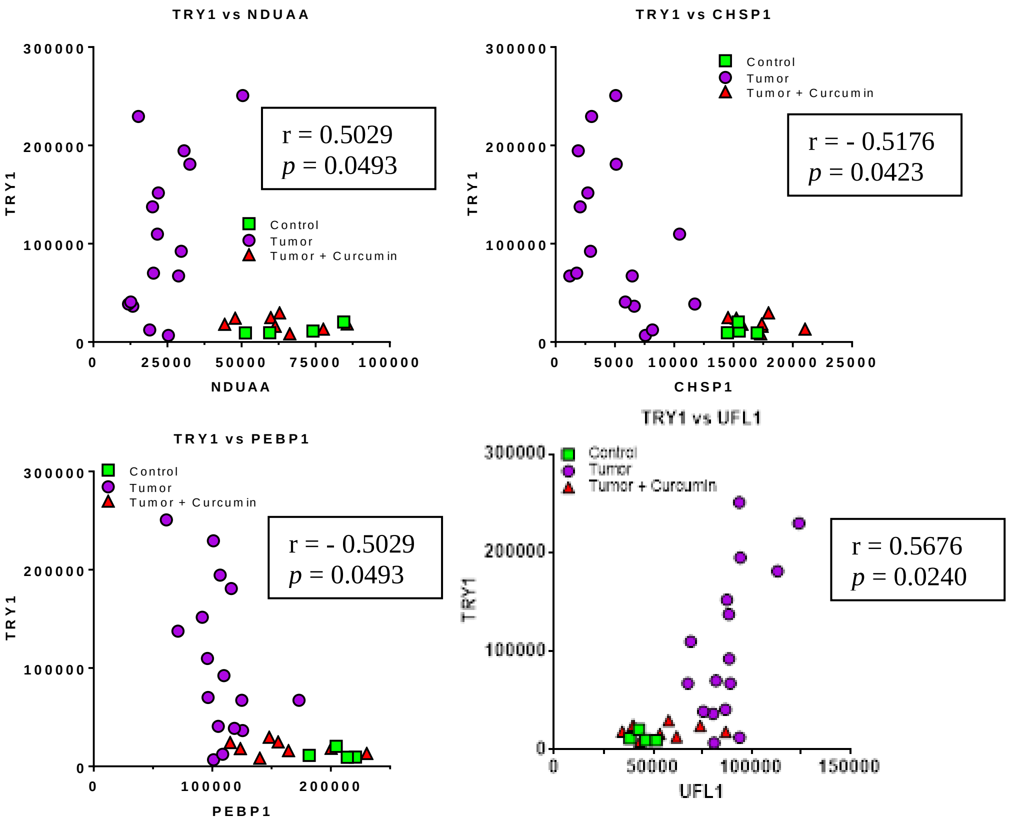

(8) Correlations with TRY3

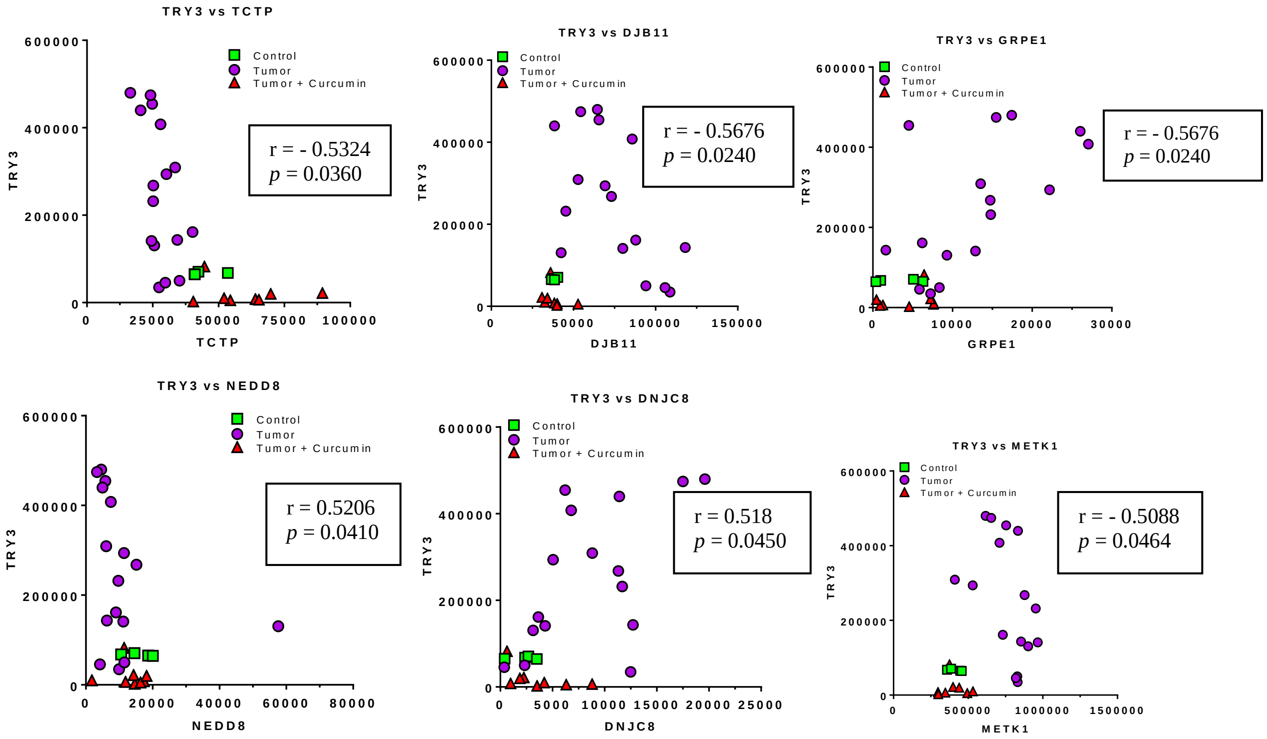

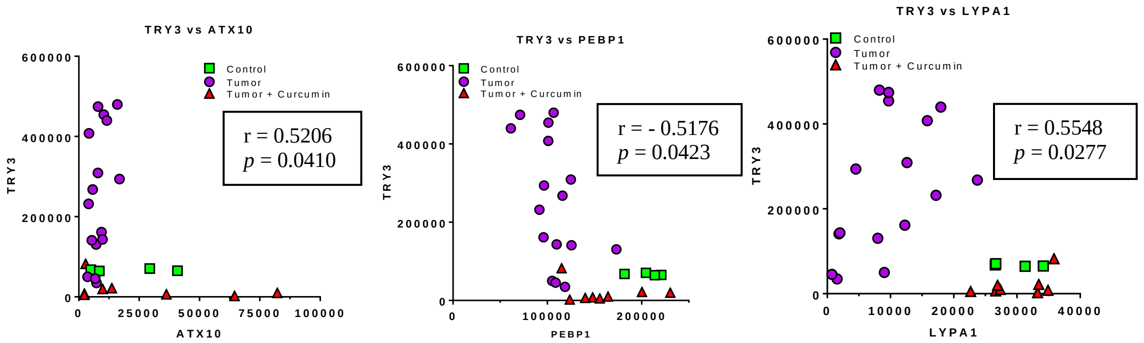

Figure S4. Correlation plots, $0.02 < p < 0.05$. Correlation plots between biomarkers identified in sections 2.2.–2.4. and 2.5. *p*- and *r*-values obtained for statistical analysis using a Spearman's test (provided only for the group of untreated rats, purple open circles) are indicated in a rectangle in each figure.

Table S1. Full names of the proteins, listing the 61 biomarkers of interest showing decreased abundance in (G2 vs. G1) and (G3 vs. G2) but not in (G3 vs. G1). The genes encoding the corresponding proteins in *homo sapiens* are provided for convenience.

Code (Rat)	Name	Gene (Human)
AATM	Aspartate aminotransferase, mitochondrial	*GOT2*
ACADM	Medium-chain specific acyl-CoA dehydrogenase, mitochondrial	*ACADM*
ACADS	Short-chain specific acyl-CoA dehydrogenase, mitochondrial	*ACADS*
ADK	Adenosine kinase	*ADK*
ADT2	ADT/ATP translocase 2	*SLC25A5*
ALDOB	Fructose-bisphosphate aldolase B	*ALDOB*
AMACR	Alpha-methylacyl-CoA racemase	*AMACR*
AMRP	Alpha-2-macroglobulin receptor-associated protein	*LRPAP1*
AP2S1	AP-2 complex subunit sigma	*AP2S1*
ARF6	ADP-ribosylation factor 6	*ARF6*
ARL2	ADP-ribosylation factor-like protein 2	*ARL2*
ARPC2	Actin-related protein 2/3 complex subunit 2	*ARPC2*
ATX10	Ataxin-10	*ATXN10*
CALM	Calmodulin	*CALM1*
CES1D	Carboxylesterase 1D	*CES1*
CHSP1	Calcium-regulated heat stable protein 1	*CARHSP1*
CO1A2	Collagen alpha-2(I) chain	*COL1A2*
COPG1	Coatomer subunit gamma-1	*COPG1*
COR1A	Coronin-1A	*CORO1A*
DECR	2,4-dienoyl-CoA reductase, mitochondrial	*DECR1*
DHRS4	Dehydrogenase/reductase SDR family member 4	*DHRS4*
ECHD3	Enoyl-CoA hydratase domain-containing protein 3, mitochondrial	*ECHDC3*
EF1D	Elongation factor 1-delta	*EEF1D*
EXOC4	Exocyst complex component 4	*EXOC4*
FIS1	Mitochondrial fission 1 protein	*FIS1*
G6PI	Glucose-6-phosphate isomerase	*GPI*
GYS2	Glycogen [starch] synthase, liver	*GYS2*
H2AZ	Histone H2A.Z	*H2AZ1/2*
HACL1	2-hydroxyacyl-CoA lyase 1	*HACL1*
HEM6	Oxygen-dependent coproporphyrinogen-III oxidase, mitochondrial	*CPOX*
HMCS2	Hydroxymethylglutaryl-CoA synthase, mitochondrial	*HMGCS2*
IDH3A	Isocitrate dehydrogenase [NAD] subunit alpha, mitochondrial	*IDH3A*
KIF3C	Kinesin-like protein KIF3C	*KIF3C*
LYPA1	Acyl-protein thioesterase 1	*LYPLA1*
MYH10	Myosin-10	*MYH10*
NCPR	NADPH—cytochrome P450 reductase	*POR*
NDUAA	NADH dehydrogenase [ubiquinone] 1 alpha subcomplex subunit 10, mitochondrial	*NDUFA10*
NEDD8	NEDD8	*NEDD8*
NEUL	Neurolysin, mitochondrial	*NLN*
ODP2	Dihydrolipoyllysine-residue acetyltransferase component of pyruvate dehydrogenase complex, mitochondrial	*DLAT*
OLA1	Obg-like ATPase 1	*OLA1*

PEBP1	Phosphatidylethanolamine-binding protein 1	*PEBP1*
PNPH	Purine nucleoside phosphorylase	*PNP*
PPAC	Low molecular weight phosphotyrosine protein phosphatase	*ACP1*
PRDX2	Peroxiredoxin-2	*PRDX2*
PRDX3	Thioredoxin-dependent peroxide reductase, mitochondrial	*PRDX3*
PRDX5	Peroxiredoxin-5, mitochondrial	*PRDX5*
PSME2	Proteasome activator complex subunit 2	*PSME2*
RAB5A	Ras-related protein Rab-5A	*RAB5A*
RAP1B	Ras-related protein Rap-1b	*RAP1B*
RINI	Ribonuclease inhibitor	*RNH1*
S10A8	Protein S100-A8	*S100A8*
SAR1B	GTP-binding protein SAR1b	*SAR1B*
SARDH	Sarcosine dehydrogenase, mitochondrial	*SARDH*
SSDH	Succinate-semialdehyde dehydrogenase, mitochondrial	*ALDH5A1*
STAT3	STAT3	*STAT3*
SYFA	Phenylalanine—tRNA ligase alpha subunit	*FARSA*
TCTP	Translationally-controlled tumor protein	*TPT1*
TPIS	Triosephosphate isomerase	*TPI1*
UFM1	Ubiquitin-fold modifier 1	*UFM1*
UGDH	UDP-glucose 6-dehydrogenase	*UGDH*

Table S2. Full names of the proteins, listing the 118 biomarkers of interest showing increased abundance in (G2 vs. G1) and (G3 vs. G2) but not in (G3 vs. G1). The genes encoding the corresponding proteins in *homo sapiens* are provided for convenience.

Code (Rat)	Name	Gene (Human)
AACS	Acetoacetyl-CoA synthetase	*AACS*
ACADL	Long-chain specific acyl-CoA dehydrogenase, mitochondrial	*ACADL*
ACON	Aconitate hydratase, mitochondrial	*ACO2*
ACPH	Acylamino-acid-releasing enzyme	*APEH*
ACTG	Actin, cytoplasmic	*ACTG1*
ACTN4	Alpha-actinin-4	*ACTN4*
ADHX	Alcohol dehydrogenase class-3	*ADH5*
AL1L1	Cytosolic 10-formyltetrahydrofolate dehydrogenase	*ALDH1L1*
AL9A1	4-trimethylaminobutyraldehyde dehydrogenase	*ALDH9A1*
AP1B1	AP-1 complex subunit beta-1	*AP1B1*
ASSY	Argininosuccinate synthase	*ASS1*
AT1A1	Sodium/potassium-transporting ATPase subunit alpha-1	*ATP1A1*
B2MG	Beta-2-microglobulin	*B2M*
CD2AP	CD2-associated protein	*CD2AP*
CERU	Ceruloplasmin	*CP*
CGL	Cystathionine gamma-lyase	*CTH*
CLH1	Clathrin heavy chain 1	*CLTC*
CNDP2	Cytosolic non-specific dipeptidase	*CNDP2*
CNN3	Calponin-3	*CNN3*
CO5A1	Collagen alpha-1(V) chain	*COL5A1*
COF1	Cofilin-1	*CFL1*
COPD	Coatomer subunit delta	*ARCN1*
CP2B1	Cytochrome P450 2B1	/
CSK	Tyrosine-protein kinase CSK	*CSK*
CTBL1	Beta-catenin-like protein 1	*CTNNBL1*
CTF8A	Chromosome transmission fidelity protein 8 homolog isoform 2	*CHTF8*
DCTN2	Dynactin subunit 2	*DCTN2*
DDB1	DNA damage-binding protein 1	*DDB1*
DDX21	Nucleolar RNA helicase 2	*DDX21*
DHCR7	7-dehydrocholesterol reductase	*DHCR7*
DHPR	Dihydropteridine reductase	*QDPR*
DHSO	Sorbitol dehydrogenase	*SORD*
DJB11	DnaJ homolog subfamily B member 11	*DNAJB11*
DNJC8	DnaJ homolog subfamily C member 8	*DNAJC8*
DX39A	ATP-dependent RNA helicase DDX39A	*DDX39A*
DX39B	Spliceosome RNA helicase Ddx39b	*DDX39B*
ECHA	Trifunctional enzyme subunit alpha, mitochondrial	*HADHA*
EF1A1	Elongation factor 1-alpha 1	*EEF1A1*
EXOC8	Exocyst complex component 8	*EXOC8*
EZRI	Ezrin	*EZR*

FABP5	Fatty acid-binding protein, epidermal	*FABP5*
FIBA	Fibrinogen alpha chain	*FGA*
FIBG	Fibrinogen gamma chain	*FGG*
FINC	Fibronectin	*FN1*
FUBP1	Far upstream element-binding protein 1	*FUBP1*
GNAI2	Guanine nucleotide-binding protein G(i) subunit alpha-2	*GNAI2*
GRB2	Growth factor receptor-bound protein 2	*GRB2*
GRPE1	GrpE protein homolog 1, mitochondrial	*GRPEL1*
GUAD	Guanine deaminase	*GDA*
H2A2A	Histone H2A type 2-A	*H2AC18*
H2AJ	Histone H2A.J	*H2AJ*
HMGN5	High mobility group nucleosome-binding domain-containing protein 5	*HMGN5*
HNRPC	Heterogeneous nuclear ribonucleoprotein C	*HNRNPC*
HNRPD	Heterogeneous nuclear ribonucleoprotein D0	*HNRNPD*
HNRPK	Heterogeneous nuclear ribonucleoprotein K	*HNRNPK*
HNRPL	Heterogeneous nuclear ribonucleoprotein L	*HNRNPL*
HS71B	Heat shock 70kDa protein 1B	*HSPA1B*
HSP7C	Heat shock cognate 71kDa protein	*HSPA8*
KIFC1	Kinesin-like protein KIFC1	*KIFC1*
KPYM	Pyruvate kinase PKM	*PKM*
LASP1	LIM and SH3 domain protein 1	*LASP1*
LTOR1	Ragulator complex protein LAMTOR1	*LAMTOR1*
MECP2	Methyl-CpG-binding protein 2	*MECP2*
METK1	S-adenosylmethionine synthase isoform type-1	*MAT1A*
MGST1	Microsomal glutathione S-transferase 1	*MGST1*
MICU1	Calcium uptake protein 1, mitochondrial	*MICU1*
ML12B	Myosin regulatory light chain 12B	*MYL12B*
MLEC	Malectin	*MLEC*
MMGT1	Membrane magnesium transporter 1	*MMGT1*
MSH2	DNA mismatch repair protein Msh2	*MSH2*
MVP	Major vault protein	*MVP*
NONO	Non-POU domain-containing octamer-binding protein	*NONO*
NSF1C	NSFL1 cofactor p47	*NSFL1C*
NUCL	Nucleolin	*NCL*
NUP53	Nucleoporin NUP53	*NUP35*
NUP93	Nuclear pore complex protein Nup93	*NUP93*
ODO1	2-oxoglutarate dehydrogenase, mitochondrial	*OGDH*
OPLA	5-oxoprolinase	*OPLAH*
PA2G4	Proliferation-associated protein 2G4	*PA2G4*
PBLD	Phenazine biosynthesis-like domain-containing protein	*PBLD*
PDLI1	PDZ and LIM domain protein 1	*PDLIM1*
PELP1	Proline-, glutamic acid- and leucine-rich protein 1	*PELP1*
PGRC1	Membrane-associated progesterone receptor component 1	*PGRMC1*
PHB	Prohibitin	*PHB*
PICAL	Phosphatidylinositol-binding clathrin assembly protein	*PICALM*
PLEC	Plectin	*PLEC*
PNO1	REVERSED RNA-binding protein PNO1	*PNO1*
PRS6A	26S protease regulatory subunit 6A	*PSMC3*
PSA7	Proteasome subunit alpha type-7	*PSMA7*
PSB1	Proteasome subunit beta type-1	*PSMB1*
PUR6	Multifunctional protein ADE2	*PAICS*
QCR8	Cytochrome b-c1 complex subunit 8	*UQCRQ*
REEP5	Receptor-expression-enhancing protein 5	*REEP5*
REEP6	Receptor-expression-enhancing protein 6	*REEP6*
ROA2	Heterogeneous nuclear ribonucleoproteins A2/B1	*HNRNPA2B1*
ROA3	Heterogeneous nuclear ribonucleoprotein A3	*HNRNPA3*
RPN1	Dolichyl-diphosphooligosaccharide—protein glycosyltransferase subunit 1	*RPN1*
RPP25	Ribonuclease P protein subunit p25	*RPP25*
RTCB	tRNA-splicing ligase Rtcb homolog	*RTCB*
S10A6	Protein S-100 A6	*S100A6*
SC31A	Protein transport protein Sec31A	*SEC31A*
SCRN1	Secernin-1	*SCRN1*
SMAD3	Mothers against decapentaplegic homolog 3	*SMAD3*
SND1	Staphylococcal nuclease domain-containing protein 1	*SND1*
SPRE	Sepiapterin reductase	*SPR*
SPTN1	Spectrin alpha-chain, non-erythrocytic 1	*SPTAN1*
SRBS2	Sorbin and SH3 domain-containing protein 2	*SORBS2*

SRSF2	Serine/arginine-rich splicing factor	*SRSF2*
SSRG	Translocon-associated protein subunit gamma	*SSR3*
STIP1	Stress-induced-phosphoprotein 1	*STIP1*
SYYC	Tyrosine—tRNA ligase, cytoplasmic	*YARS1*
TMM43	Transmembrane protein 43	*TMEM43*
TRY1	Anionic trypsin-1	*PRSS1*
TRY3	Cationic trypsin-3	*PRSS3*
UBA1	Ubiquitin-like modifier-activating enzyme 1	*UBA1*
UFL1	E3 UFM1-protein ligase 1	*UFL1*
VDAC2	Voltage-dependent anion-selective channel protein 2	*VDAC2*
VIME	Vimentin	*VIM*
YBOX1	Nuclease-sensitive element-binding protein 1	*YBX1*

Table S3. Changes in liver protein abundances within the G2 group (Advanced vs Initial stages of M5-T1 tumor development).

Protein Code (Rat)	Initial Stage, Median	Advanced Stage, Median	*p* value
AACS	*54067*	*40564*	*ns (0.3282)*
AATM	*622927*	*757173*	*ns (0.2345)*
ACADL	*524220*	*502495*	*ns (0.3823)*
ACADM	*44848*	*35433*	*ns (0.7209)*
**ACADS**	**111467**	**27651**	**0.0379**
ACON	*168882*	*160882*	*ns (0.3823)*
ACPH	*90423*	*73123*	*ns (0.3282)*
ACTG	*3081679*	*3240086*	*ns (0.7984)*
ACTN4	*69646*	*75790*	*ns (0.9591)*
**ADHX**	**26754**	**36789**	**0.0379**
ADK	*5405*	*6606*	*ns (0.5054)*
ADT2	*55429*	*55796*	*ns (0.5737)*
**AL1L1**	**668368**	**893241**	**0.0499**
AL9A1	*1192309*	*996066*	*ns (0.1049)*
ALDOB	*1983863*	*1876886*	*ns (0.7209)*
AMACR	*10162*	*14510*	*ns (0.3282)*
AMRP	*3792*	*2500*	*ns (0.8785)*
AP1B1	*11770*	*15779*	*ns (0.2345)*
**AP2S1**	**12758**	**25707**	**0.0281**
APOA4	*12433*	*22665*	*ns (0.2786)*
ARF6	*2841*	*3277*	*ns (0.7984)*
**ARL2**	**4038**	**21901**	**0.0499**
ARPC2	*28296*	*33716*	*ns (0.2345)*
ASSY	*57872*	*97565*	*ns (0.1049)*
AT1A1	*129314*	*128039*	*ns (>0.9999)*
ATX10	*7233*	*7960*	*ns (0.5737)*
B2MG	*31821*	*32615*	*ns (0.8785)*
CALM	*23562*	*22814*	*ns (0.5737)*
CD2AP	*33603*	*27689*	*ns (0.5054)*
CERU	*177331*	*209497*	*ns (0.6454)*
CES1D	*204844*	*275233*	*ns (0.1049)*
CGL	*2013692*	*1848823*	*ns (0.5737)*
CH60	*311063*	*238775*	*ns (0.2786)*
**CHSP1**	**7101**	**2880**	**0.0499**
CLH1	*195514*	*280420*	*ns (0.1049)*
CNDP2	*103366*	*101595*	*ns (0.9591)*
CNN3	*15879*	*12804*	*ns (0.8785)*
CO1A2	*30129*	*18045*	*ns (0.3282)*
COF1	*134527*	*118344*	*ns (0.1949)*
COPD	*9097*	*5741*	*ns (0.4418)*
COPG1	*9050*	*10730*	*ns (0.9591)*
COR1A	*10541*	*7114*	*ns (0.5054)*
CSK	*49268*	*28186*	*ns (0.3823)*
CTBL1	*6017*	*10129*	*ns (0.1949)*
CTF8A	*8446*	*9105*	*ns (0.9591)*
DCTN2	*46494*	*48093*	*ns (0.5054)*
DDB1	*11408*	*15120*	*ns (0.7984)*
DDX21	*20668*	*19167*	*ns (0.8785)*
DECR	*146429*	*147510*	*ns (0.7984)*
DHCR7	*130389*	*116207*	*ns (0.2786)*
DHPR	*215100*	*280203*	0.0650
DHRS4	*11940*	*13738*	*ns (0.5737)*
DHSO	*1650070*	*1924315*	*ns (0.2345)*
**DJB11**	**91021**	**59473**	**0.0281**
**DNJC8**	**3970**	**11361**	**0.0499**
**DX39A**	**14813**	**35155**	**0.0207**
DX39B	*57970*	*61151*	*ns (0.7209)*

**ECHA**	**648835**	**358761**	**0.0002**
ECHD3	*174101*	*149514*	*ns (0.3823)*
EF1A1	*3235568*	*2920253*	*ns (0.3823)*
EF1D	*39285*	*49138*	*ns (0.1304)*
EXOC4	*1582*	*5741*	*ns (0.1049)*
EXOC8	*21379*	*17401*	*ns (0.5737)*
EZRI	*39159*	*37765*	*ns (0.7209)*
**FABP5**	**13027**	**3054**	**0.0003**
**FETUA**	**60527**	**102185**	**0.0148**
FIBA	*200601*	*314166*	*ns (0.2345)*
**FIBG**	**175924**	**313436**	**0.0104**
**FINC**	**282832**	**172271**	**0.0207**
FIS1	*13323*	*8000*	*ns (0.3282)*
FUBP1	*13730*	*19747*	0.0830
G6PI	*36406*	*34066*	*ns (0.8785)*
GNAI2	*55029*	*67654*	*ns (0.7984)*
GRB2	*11393*	*11318*	*ns (0.6454)*
GUAD	*167200*	*146823*	*ns (0.1949)*
GYS2	*1828*	*2223*	*ns (0.6454)*
H2A2A	*9164*	*10380*	*ns (0.2345)*
**H2AJ**	**66357**	**29515**	**0.0499**
H2AZ	*14744*	*14851*	*ns (0.3823)*
HA12	*40113*	*16492*	0.0830
HACL1	*24953*	*12628*	*ns (0.7984)*
HEM6	*18652*	*11635*	*ns (0.5737)*
**HMCS2**	**751183**	**1204017**	**0.0281**
HMGN5	*17418*	*6496*	*ns (0.1304)*
HNRPC	*159042*	*138998*	*ns (0.2345)*
HNRPD	*55780*	*54977*	*ns (>0.9999)*
**HNRPK**	**112930**	**83132**	**0.0499**
HNRPL	*17842*	*20386*	*ns (0.8785)*
HS71B	*1043077*	*1078850*	*ns (0.8785)*
HSP7C	*499867*	*478945*	*ns (0.4418)*
ICAM1	*10653*	*15469*	*ns (0.1949)*
**IDH3A**	**6822**	**19339**	**00.0281**
IL6RB	*12089*	*22008*	*ns (0.1605)*
**IRGM**	**3728**	**6088**	**0.0148**
KIF3C	*3502*	*5543*	*ns (>0.9999)*
KIFC1	*2386*	*1914*	*ns (>0.9999)*
KPYM	*44799*	*36281*	*ns (0.3823)*
LASP1	*63529*	*54398*	*ns (0.1605)*
LTOR1	*30081*	*31053*	*ns (0.8785)*
**LYPA1**	**4995**	**14210**	**0.0115**
MECP2	*3153*	*2773*	*ns (0.6454)*
METK1	*829946*	*681863*	*ns (0.1605)*
MGST1	*17786*	*8320*	*ns (>0.9999)*
MICU1	*909*	*1928*	*ns (0.3823)*
MIF	*312747*	*450410*	*ns (0.5054)*
ML12B	*175875*	*135185*	*ns (0.1605)*
MLEC	*52041*	*58759*	*ns (0.4418)*
**MMGT1**	**6133**	**11601**	**0.0499**
MSH2	*4342*	*7815*	*ns (0.3282)*
MVP	*72490*	*43848*	*ns (0.1605)*
MYH10	*8826*	*7906*	*ns (0.6454)*
**NCPR**	**37874**	**75296**	**0.0104**
NDUAA	*20313*	*25286*	*ns (0.1605)*
NEDD8	*7644*	*6770*	*ns (0.7984)*
NEUL	*7502*	*6908*	*ns (0.8785)*
NONO	*70718*	*68258*	*ns (0.4418)*
NSF1C	*107595*	*103870*	*ns (0.7984)*
NUCL	*48934*	*51716*	*ns (0.8785)*
NUP53	*10692*	*7854*	*ns (0.6454)*
NUP93	*29848*	*23947*	*ns (0.4418)*
ODO1	*120449*	*100847*	*ns (0.5242)*
ODP2	*156743*	*113127*	*ns (0.1949)*
OLA1	*11061*	*12810*	*ns (0.8785)*
OPLA	*34623*	*30264*	*ns (0.9591)*
**PA2G4**	**88770**	**65574**	**0.0281**
PBLD	*118238*	*131904*	*ns (0.1049)*
**PDLI1**	**30453**	**74575**	**0.0499**
PEBP1	*109333*	*98742*	*ns (0.1049)*
**PELP1**	**3825**	**8821**	**0.0379**
PHB	*126195*	*122604*	*ns (0.8785)*
**PICAL**	**62476**	**30687**	**0.0070**
PLEC	*63708*	*48306*	*ns (0.2786)*
PNO1	*956*	*2024*	*ns (0.2345)*
PNPH	*108669*	*156942*	*ns (0.1049)*

**PPAC**	**27304**	**40260**	**0.0499**
PRDX2	*127612*	*158085*	*ns (0.3282)*
PRDX3	*36289*	*36075*	*ns (0.9591)*
PRDX5	*223549*	*252387*	*ns (0.7209)*
PRS6A	*57537*	*47312*	*ns (0.1605)*
PSA7	*22612*	*18462*	*ns (0.7984)*
PSB1	*101503*	*117910*	*ns (0.5054)*
PSME2	7986	10174	0.0830
PUR6	*39544*	*41576*	*ns (0.7984)*
QCR8	*31395*	*36071*	*ns (0.8785)*
RAB5A	*18042*	*20896*	*ns (0.2345)*
RAP1B	*31999*	*46501*	*ns (0.1049)*
REEP5	*1230*	*1403*	*ns (0.6454)*
REEP6	*255248*	*264586*	*ns (0.1949)*
RINI	*8313*	*13660*	*ns (0.2786)*
ROA2	*214734*	*193410*	*ns (0.1049)*
ROA3	*64200*	*68160*	*ns (0.6454)*
RPN1	*767581*	*771925*	*ns (0.7209)*
RPP25	*5711*	*9637*	*ns (0.2345)*
**RTCB**	**47414**	**66022**	**0.0499**
S10A4	*19119*	*12580*	*ns (0.3823)*
S10A8	*3316*	*3791*	*ns (0.2906)*
S10AB	6536	11120	0.0650
SAR1B	*48285*	*42590*	*ns (0.7209)*
SC22B	*110489*	*96745*	*ns (0.7209)*
SC31A	*70981*	*74238*	*ns (0.5737)*
SCRN1	*2061*	*2904*	*ns (0.7984)*
SMAD3	*13351*	*10730*	*ns (0.5054)*
SND1	*432306*	*443993*	*ns (0.6454)*
SPRE	*188791*	*161003*	*ns (0.5737)*
SPTN1	*71821*	*55255*	*ns (0.5737)*
SRBS2	*33983*	*14806*	*ns (0.1049)*
**SRSF2**	**96083**	**59908**	**0.0499**
SSDH	*5317*	*16903*	*ns (0.1605)*
SSRG	*85094*	*123978*	*ns (0.6943)*
STAT3	*3245*	*6471*	*ns (0.1049)*
STIP1	*71664*	*65649*	*ns (0.3282)*
SYFA	22306	15545	0.0830
SYYC	*37680*	*38234*	*ns (0.7209)*
TAP2	*3806*	*8174*	*ns (0.1949)*
TCTP	*28580*	*25202*	*ns (0.1605)*
TMM43	*12055*	*20745*	*ns (0.1949)*
TPIS	*59558*	*34512*	*ns (0.1304)*
UBA1	*147682*	*132338*	*ns (0.2786)*
UFL1	*83963*	*91275*	*ns (0.1949)*
UFM1	*4832*	*6985*	*ns (0.3282)*
UGDH	*103446*	*99347*	*ns (0.7209)*
VDAC2	*109319*	*115990*	*ns (0.5737)*
**VIME**	**610917**	**225269**	**0.0104**
YBOX1	*6932*	*6409*	*ns (0.8785)*

The list includes all proteins (in alphabetical order of their code). described in Figures 2 to 7, full names being indicated in the text or Tables S1 and S2. Mann-Whitney statistical test. For all non-significant difference (*ns*), medians and *p* values are indicated in italics. Significant differences are indicated in bold type, and tendencies in normal type. Proteins showing more pronounced decrease or increase in link with tumor progression are additionally highlighted in yellow.

© 2020 by the authors. Licensee MDPI, Basel, Switzerland. This article is an open access article distributed under the terms and conditions of the Creative Commons Attribution (CC BY) license (http://creativecommons.org/licenses/by/4.0/).
